# Expression of Genes Related to Sugar and Amino Acid Transport and Cytokinin Metabolism during Leaf Development and Senescence in *Pisum sativum* L.

**DOI:** 10.3390/plants8030076

**Published:** 2019-03-25

**Authors:** Annu S. Ninan, Jan Grant, Jiancheng Song, Paula E. Jameson

**Affiliations:** 1 School of Biological Sciences, University of Canterbury, Private Bag 4800, Christchurch 8140, New Zealand; annu.ninan@canterbury.ac.nz (A.S.N.); jsong@ytu.edu.cn (J.S.); 2 The New Zealand Institute for Plant & Food Research Limited, Private Bag 4704, Christchurch 8140, New Zealand; jan.grant@plantandfood.co.nz; 3 School of Life Sciences, Yantai University, Yantai 264005, China

**Keywords:** amino acid transporter (*AAP*), cytokinin, cytokinin oxidase, isopentenyl transferase, pea, senescence, source–sink, sucrose transporter (*SUT*), *SWEET*

## Abstract

Gene editing is becoming the plant breeding tool of choice, but prior to targeting a gene for editing, a knowledge of the gene family members (GFMs) controlling yield is required in the specific crop plant. Critical to yield are components from senescing leaves. We targeted genes controlling senescence in *Pisum sativum* and the release and transport of carbohydrates and amino acids from the source leaves to the pods and seeds. The expression of GFMs for cytokinin biosynthesis (*IPT*) and destruction (*CKX*), sucrose transporters (*SUT*), Sugar Will Eventually be Exported Transporters (*SWEET*), amino acid permeases (*AAP)*, and cell wall invertases, was determined using RT-qPCR. GFMs were differentially expressed in leaves of different ages. The expression of many gene family members was lower in the expanding sink leaves compared with the senescing leaves, with the exception of two *PsAAP* GFMs and *PsCKX5*, which were highly expressed. Expression of specific *PsSWEET*s, *SUT*s, and *AAP*s increased in the mature and/or senescing leaves. Expression of *PsIPT*s was least in the mature source leaves, but as strong in the senescing leaves as in the young source leaves. *PsCKX7* was expressed in source and senescing leaves. We discuss the potential impact of the targeted reduction of specific *PsCKX* GFMs on source-sink relationships.

## 1. Introduction 

For agronomic plants, a critical component that contributes to both yield and nutritional content is the duration of the leaf’s life span and its ultimate senescence [1]. During its life span, the typical developing leaf is initially a sink organ—drawing on the resources of the parent plant. During the phase of rapid expansion, the leaf changes to become a source organ, supplying carbohydrate to the plant. Eventually, all leaves undergo senescence. During this process cellular constituents are dismantled, and metabolites are actively recycled and exported from the leaf [1,2]. The sink for these metabolites varies depending on the life cycle of the plant. For annual legumes, the pod and developing seeds are the key sink tissues. Legumes have been identified as being of increasing importance in sustainable food production and for human health benefits [3] and, while leaf senescence in *Pisum sativum* L. (pea) has been described [4], little is known about the expression of the key genes in the pea leaf which co-ordinate the supply of carbohydrate and amino acids to the developing pod and seeds.

We know from transcriptomic data that the expression of multiple genes changes during the onset and progression of senescence—the so-called senescence associated genes (*SAG*s) [5,6,7]. There is a tightly regulated dismantling of intracellular structures (starting with the chloroplasts) in addition to the breakdown of proteins, nucleic acids and lipids, and the mobilisation of sugars and amino acids [8]. The energy required for the progression of senescence is supplied by the continued presence of mitochondria late into the process [9]. 

Since the 1960s, cytokinins have been implicated in the enhancement of sink activity in leaves [10] and in the delay of senescence [11], with the cytokinin-induced delay of senescence shown to be dependent on the activity of cell wall invertases (CWINV) [12]. The key step in cytokinin biosynthesis is controlled by isopentenyl transferase (IPT), while that of cytokinin degradation is controlled by cytokinin oxidase/dehydrogenase (CKX). Both enzymes are encoded by gene families, including in pea [13,14]. Early transcriptomic analyses during the developmental leaf senescence of *Arabidopsis thaliana* [5] showed decreased expression of an *AtIPT* and of cytokinin response regulators (*RRs*) and increased expression of an *AtCKX.* Others have shown decreases in active forms and increases in inactive forms of endogenous cytokinins in senescing leaves of a variety of species [15,16,17]. However, after a comprehensive analysis of gene expression and endogenous cytokinins, Edlund et al. [18] suggested that the depletion of cytokinin was unlikely to explain the onset of autumn leaf senescence in aspen, lending support to the statement by Jibran et al. [19] that cytokinin affects the process of senescence but not the regulation of the onset of senescence.

Loading of sugars from source and senescing leaves into the phloem requires the co-ordinated activity of the recently identified Sugar Will Eventually be Exported Transporters (SWEETs) and the sucrose transporters (SUTs) [20,21]. Individual *SWEET* gene family members (GFMs) have been identified in critical roles for plant development and plant–pathogen interactions [22]. SWEETs belonging to Clades I, II, and IV transport hexoses, while those belonging to Clade III transport sucrose [23,24,25]. Clade IV SWEETs transport hexoses to the vacuole, while members of Clades I, II, and III transport sugars across the plasma membrane to the apoplast [26]. The activity of SWEETS over the lifetime of a leaf has not been shown for many species. Greater expression of *SWEETS* in source leaves compared with young sink leaves has been shown for cucumber [25] and for *SWEET10* in sweet potato [26], while *SAG29*—subsequently renamed as Clade III *AtSW15*—was shown to increase markedly in senescent Arabidopsis leaves [27]. Overexpression of both *AtSW15* [27] and of Clade II rice *OsSW5* [28] resulted in precocious senescence. Bezrutczyk et al. [29] report impaired phloem loading in maize in which *ZmSW13a,b,c* were knocked out. *PsSWEET* gene family members have been shown to be expressed in the germinating seedling [30] and to be responsive to both epiphytic and endophytic *Rhodococcus fascians* [13].

Young expanding leaves are regarded as sinks for nitrogen, with N transporters effecting uptake of N into the leaves from the xylem and phloem, whereas mature and senescing leaves are sources of N, utilising transporters to export N into the phloem for transport to sinks [20]. N uptake and assimilation has been closely linked to the activity of cytokinins [31,32]. Enhancing endogenous cytokinin has been shown to delay senescence [33,34,35], as well as the release of N from senescing leaves [36,37,38]. As the amino acid permease (AAP) transporters operate on the phloem pathway throughout the plant [20], we monitored the expression of AAP gene family members in pea leaves.

A key breeding target has been identified as the manipulation of source/sink relationships during pod and seed development in legumes [39,40,41,42]. Plant breeding using molecular methods is dependent on the knowledge of the differential activity of individual gene family members throughout the plant, particularly if molecular tools such as gene editing are to be utilised. We have shown that family members of transporter and cytokinin biosynthesis and degradation genes express differentially—both spatially and temporally—during the germination of pea seeds [30] and pre- and post-fertilisation in pods and seeds [14], but little is known about the expression of these genes during leaf development and senescence in peas. We targeted those genes controlling senescence and the release and transport of carbohydrates and amino acids from the source leaves to the pods and seeds.

## 2. Results

We used two popular breeding cultivars of pea, Bolero, a process pea and Bohatyr, a “field” pea as biological replicates to determine which gene family members are consistently expressed despite different breeding histories and growth strategies. Bolero is a “process” pea cultivar with a vining growth habit, with pods harvested immature for both fresh and frozen peas, whereas Bohatyr is a conventional leafed “field” pea, harvested when mature for its protein content as a dried pea. The data are presented as the combined average of the two cultivars, with the data for the individual cultivars shown in supplementary materials.

### 2.1. Chlorophyll Analyses

**Figure 1:**
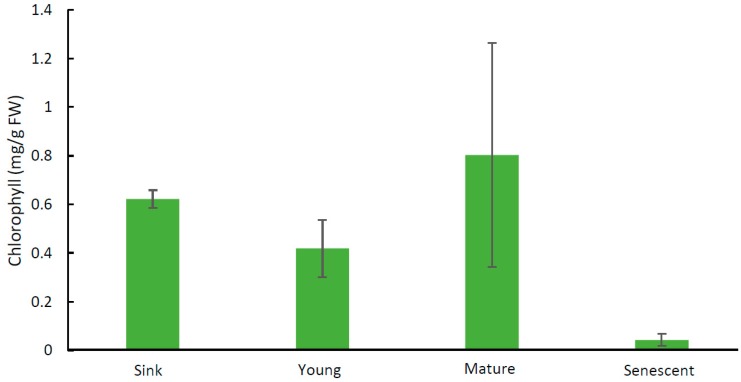
Chlorophyll content of leaves of *Pisum sativum*. Expanding sink leaves (sink), source leaves subtending pods five days after flowering (young) and 20 days after flowering (mature), and senescing leaves were analysed. Data are the averages of two biological replicates, with three replicate extractions for each sample. Results are ± s.d.

The chlorophyll content was similar in the expanding sink leaves and the source leaves supporting pods 5 and 20 days after flowering, and thereafter decreased as the leaves began senescing (Figure 1).

### 2.2. Gene Expression

**Figure 2:**
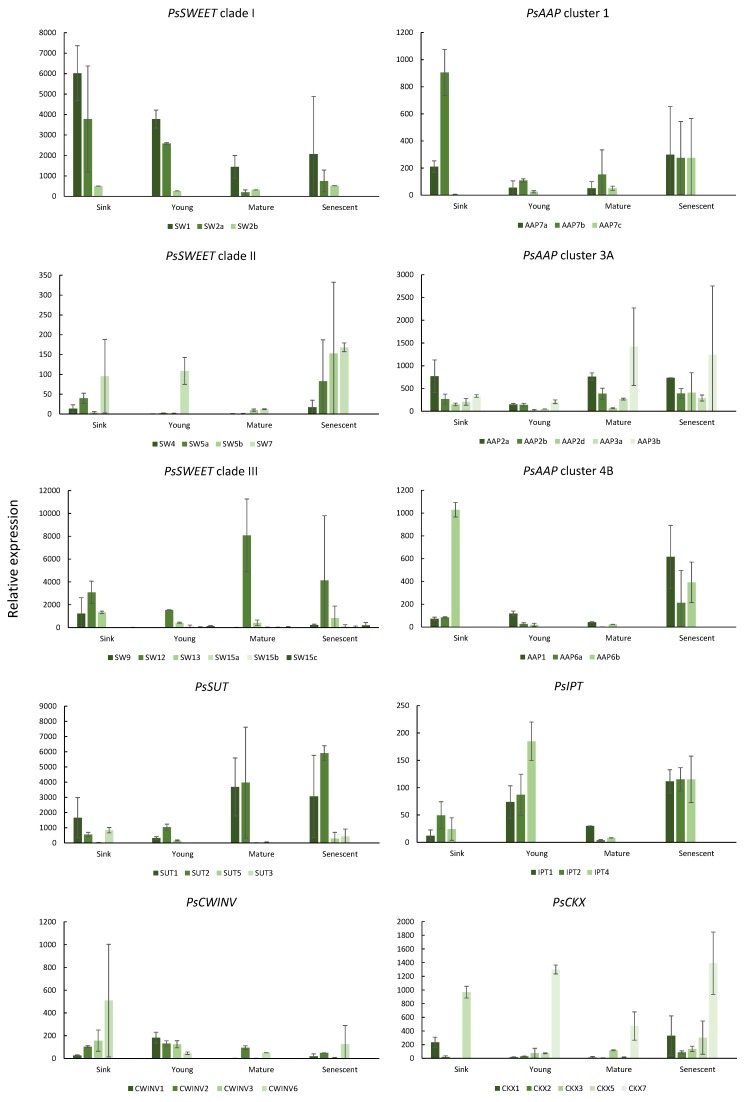
Relative expression of Sugar Will Eventually be Exported Transporter (*SWEET*), sucrose transporter (*SUT*), amino acid permease (*AAP*) transporter gene families, cell wall invertase (*CWINV*), cytokinin biosynthesis (*IPT*), and cytokinin degradation (*CKX*) gene families in leaves of *Pisum sativum* of different ages (sink: expanding sink leaves; young: source leaves subtending pods five days after flowering; mature: source leaves subtending pods 20 days after flowering; senescent: senescing leaves). Mean fold-change values (y-axis) were calculated using *PsEF*, *PsGAP*, and *PsACT* as internal controls. The results are expressed as the average of two biological replicates, with three technical replicates for each. Results are ± s.d.

Expression data are shown in Figure 2. While there was expression of several Clade I *SWEET*s in leaves of different ages, there was increased expression of several Clade III *SWEET*s in the mature and senescing leaves. Expression of *PsSUT*s *1* and *2* increased as the leaves aged. Expression of the *PsCWINV*s was generally lowest in the mature and/or senescing leaves.

*PsAAP6b* and *7b* showed strong expression in the expanding sink leaves. The majority of the *PsAAP* gene family members showed the least expression in the young source leaves, while increased expression of several gene family members was detected in the mature source leaves. Many of the *PsAAP* gene family members were expressed strongly in the senescing leaves, the exception being Cluster 4B *PsAAP8,* which showed little expression in leaves of any age (data not shown).

The three *PsIPT* gene family members expressed in the young source leaves showed decreased expression in the mature source leaves but increased expression again in the senescent leaves. A similar pattern was shown for *PsCKX* expression, but with *PsCKX5* expressed in the sink leaves, and *PsCKX7* in the source and senescing leaves.

## 3. Discussion

For translocation through the plant, sucrose must be moved across the plasma membrane to the apoplast and thence into the phloem. In Arabidopsis, Clade III SWEET11 and 12 have been identified as controlling the first step in loading sucrose into the phloem for long-distance transport [24]. Clade III SWEETs load sucrose into the apoplast, from where it is loaded by SUTs into the phloem [22,24]. Previously, an individual *SWEET* Clade III gene family member (*AtSW15*; *SAG29*) was identified as showing increased expression in senescing leaves of Arabidopsis and accelerated senescence in over-expressed lines [27]. In pea, we showed a notable increase in expression in several Clade II and III *SWEET*s in the senescing leaves. A senescing leaf is offloading its carbohydrate, and the increased expression of the *SWEET*s was matched by increased expression of *PsSUT1* and *2* as the leaves aged. A similar coincidence of expression between *SWEET*s and *SUT* transporters occurs in various tissues of *Brassica napus* L. [21].

*PsCWINV* family members showed differential expression, as also shown in *B. napus* leaves [43]. Expression of *PsCWINV*s may have led to increased hexoses in the expanding leaves, providing a strong osmoticum to support expansion. Strong expression of specific *PsAAP* gene family members during leaf expansion is indicative of the import of amino acids into the developing sink leaves [44]. Several *PsAAP*s were expressed in the mature source and senescing pea leaves. However, the expression of *PsAAP8* was not detected (data not shown), even though *AtAAP8* has been classified as “the long sought after phloem loader” [45] in Arabidopsis. *AAP8* belongs to Cluster 4B, the cluster that *PsAAP1* and *6* are allocated to. However, we showed that it is members of Cluster 3A which were most highly expressed in the mature source leaves of pea. Not surprisingly, the *PsAAP*s were strongly expressed in the senescing leaves, as these AAPs aid in the export of the degraded protein as the leaf senesces [8]. Moreover, during pea leaf senescence the transport of amino acids was not delegated to any specific cluster. Similar patterns are seen also for *B. napus AAP* gene family members [43,46].

Low expression of *PsIPT* gene family members in the sink leaves was not unexpected, and has been shown in both brassicas [43,47] and cereals [48]. Xylem-supplied cytokinin moves readily with the transpiration stream and accumulates in sink leaves [49], indicating that root-supplied cytokinin potentially provides all necessary cytokinin to the expanding sink [50]. The decrease in *PsIPT* expression in the mature source leaves during pod filling is not surprising either, as the release of nutrients is required, rather than a cytokinin-enhanced sink activity [10]. According to the data from Buchanan-Wollaston et al. [5], an increase in expression of *PsIPT* in senescing leaves would not have been expected, although an increase has been reported before in both rapid cycling *Brassica rapa* [51], and forage brassica *B. napus* [43], and an increase in the cytokinin-activating gene, *LOG7a*, was detected in senescing aspen leaves [18]. Additionally, both van der Graff et al. [6] and Edlund et al. [18] showed upregulation of specific cytokinin response regulators in senescing leaves of Arabidopsis and aspen, respectively. However, in pea, there appears to be tight regulation of any additional cytokinin (either arriving via the xylem or newly biosynthesized), as increased activity of *PsCKX5* occurred in the sink leaves, and increased expression of *PsCKX7* occurred in the maturing and senescing leaves. Interestingly, *CKX7* was also specifically upregulated in senescing aspen leaves [18], as were several *BnCKX* gene family members [43].

It is possible that the role of cytokinins as scavengers of reactive oxygen species (ROS) is required in senescing leaves. Cytokinin can enhance the activities of anti-oxidant enzymes such as catalase, superoxide dismutase, peroxidase, and ascorbate peroxidase [52,53,54,55,56]. Reactive oxygen species are produced both in the chloroplast [53] and in the mitochondria [57] and can be damaging to membrane integrity [55]. We suggest that cytokinin may be required to assist the maintenance of mitochondrial integrity until the very final stages of senescence through the activation of leaf anti-oxidant activities. Besides providing energy during leaf senescence, the mitochondria play a key role in nitrogen remobilisation [9]. However, any role for cytokinin during the active phase of senescence is likely to be complex [6,18].

*PsCKX7* was expressed in the young, mature, and senescing leaves. *PsCKX7* was also expressed in the meristem of peas, in the pea ovule one day before fertilisation [14], and during early shoot and root growth of pea seedlings [30]. A deficiency in cytokinin has been suggested as a causal factor in limiting the pod number in legumes [58], and the downregulation of *PsCKX7* in the shoot meristem has been suggested as a target for gene editing, or for Targeted Induced Loci Lesions IN Genomes (TILLING) [14]. Downregulation of *PsCKX7* may also lead to increased cytokinin in the shoots, roots, and leaves. Modestly increased endogenous cytokinin has been shown to enhance stress tolerance [35]. Pea yield is reported to be affected by stress and particularly by high temperatures [59]. Savada et al. [60] suggest that the effect of heat stress may be through ethylene biosynthesis and signalling. As there is a complex interaction between cytokinin and the perception and/or biosynthesis of ethylene [61], enhancing stress tolerance in pea—particularly at the time of pollination and fruit set—via elevated levels of cytokinin would need to be carefully evaluated. Delayed leaf senescence may be another consequence of the downregulation of *PsCKX7*. Whether this is beneficial to the yield and protein content of seeds will depend on the resulting strength of the mature and senescing leaves in retaining metabolites [36].

In summary, detailed knowledge of the temporal specificity of gene family members within cultivars of a species is not only useful but required before being able to target specific transporter or cytokinin gene family member(s) for gene editing in order to avoid unintended effects.

## 4. Materials and Methods

### 4.1. Plant Material

Seeds of *P. sativum* L. cv. Bolero (a process pea) and cv. Bohatyr (a field pea) were sourced from The New Zealand Institute for Plant & Food Research. Both cultivars represent the industry standard and are pure lines. Twenty-two seeds of each line were sown (planted) during summer (December, Christchurch, New Zealand). Each seed was sown in a pot containing potting mix, mixed with fertilizers. Growth room conditions were 22 °C during the 16 h day and 14 °C during the 8 h night. Plants were watered every two days to keep the plants moist but not wet. Plants were fertilised two or three times during the growing period. Flowers were tagged just as the standard petal opened.

Plant material for gene expression and chlorophyll analysis was obtained from pooled samples of individual leaves collected from five cv. Bolero and five cv. Bohatyr plants. The leaf samples collected were young expanding sink leaves, source leaves, and senescent leaves. Two sets of source leaves were collected: those supporting pods five days after flowering (DAF) and those supporting pods 20 DAF. Both types of leaves were fully expanded, but those associated with pods 5 DAF are here referred to as “young source leaves” and those collected when pods were 20 DAF are referred to as “mature source leaves”. Leaf samples were collected into 15 mL falcon tubes, immediately frozen in liquid nitrogen, and stored at −80 °C.

### 4.2. Gene Isolation and Sequence Analysis

Sequences of family members from candidate genes of interest were isolated from an RNA-Seq transcriptome as described in Dhandapani et al. [13]. A pool of total RNA samples extracted separately from multiple developmental stages of leaves, flowers, and siliques was used to construct the cDNA library, which was then sequenced using an Illumina HiSeq2000 genome analyser at the Beijing Genomic Institute (BGI) customer service. Orthologue sequences of *IPT*, *CKX*, *SUT*, *AAP*, *CWINV*, and *SWEET* from legume species available in the GenBank database were used as query sequences to BLAST search our *P. sativum* transcriptome using prfectBLAST 2.0. The putative sequences were verified via BLAST searching the GenBank database and multiple sequence alignment with representative orthologue sequences in closely related species [13].

### 4.3. RNA Isolation and cDNA Synthesis

Total RNA was extracted from up to 100 mg of frozen samples using TRIzol Reagent (Invitrogen, Carlsbad, CA, USA) following the manufacturer’s instructions and immediately stored at −20 °C. The integrity and quality of isolated RNA was assessed by running 1 μL samples on a 1% (w/v) agarose gel. The concentration and purity of the total RNA was assessed using a Nanodrop™ spectrophotometer. Extracted RNA was converted to cDNA through reverse transcription. Approximately 1 µg of total RNA, 50U Expand Reverse Transcriptase (Roche, Mannheim, Germany), 50 pmol oligo (dT) primers and 100 pmol random hexamer (pdN6) primers were used in a 20 μL reaction. The final reaction mix was incubated at 42 °C for 2 h, and then at 70 °C for 15 min to deactivate the enzyme. The cDNA was diluted 10-fold with nanopure water and stored at −20 °C.

### 4.4. Quantitative Reverse Transcription Polymerase Chain Reaction

Quantitative RT-PCR was used to measure relative gene expression of the individual family members across the various plant tissues as they developed. Specific PCR primers were designed for each family member of the five genes of interest. A volume of 15 µL was used for all qPCR reactions in a Rotor-Gene Q (Qiagen) real-time PCR instrument, using home-made SYBR Green master mix or a KAPA SYBR^®^ FAST qPCR Kit (Kapa Biosystems, Boston, USA). Three reference genes, elongation factor (*EF*), actin (*ACT*) and *GAPDH*, were used as internal controls to normalise the data by correcting for differences in the quantity of cDNA used as templates, as described previously [13,14,62]. The relative expression (fold change) of each target gene was then calculated using the 2^−ΔΔ^C_t_ method [63] using a constant ΔC_t_ of 30 minus the average correction factor derived from the reference genes as the value against which all samples were calculated. Three technical replicates for each of the two biological replicates (Bolero and Bohatyr) were carried out for each sample set.

### 4.5. Chlorophyll Estimation

Chlorophyll from three sub-samples of the pooled leaves for each developmental stage was extracted into DMF and analysed using a Nanodrop spectrophotometer as described in Evans et al. [64]. The total chlorophyll content was calculated using the equation of Wellburn [65]: Chl_total_ = 7·12*A*_664_ + 18·12*A*_647._

## Supplementary Materials

The following are available online at https://www.mdpi.com/2223-7747/8/3/76/s1, Figure S1: Chlorophyll content of leaves of *Pisum sativum* cvs. Bolero and Bohatyr. Figure S2: Relative expression of *PsSWEET*, *SUT* and *AAP* transporter gene families, *CWINV*, cytokinin biosynthesis (*IPT*) and cytokinin degradation (*CKX*) gene families in leaves of *Pisum sativum* cv. Bolero of different ages. Figure S3: Relative expression of *PsSWEET*, *SUT*, and *AAP* transporter gene families, *CWINV*, cytokinin biosynthesis (*IPT*) and cytokinin degradation (*CKX*) gene families in leaves of *Pisum sativum* cv Bohatyr of different ages.
