# Supplementary figures and images for: Expression of Genes Related to Sugar and Amino Acid Transport and Cytokinin Metabolism during Leaf Development and Senescence in Pisum sativum L
Source: Plants (Basel). 2019 Mar 25;8(3):76. doi: 10.3390/plants8030076 (PMC6473372; doi:10.3390/plants8030076)

Relative expression (mean fold change)

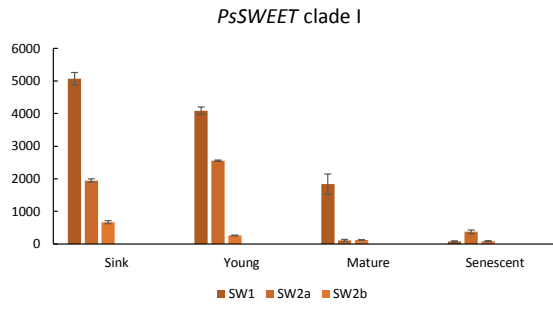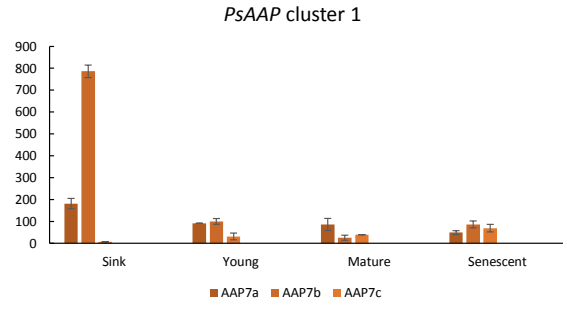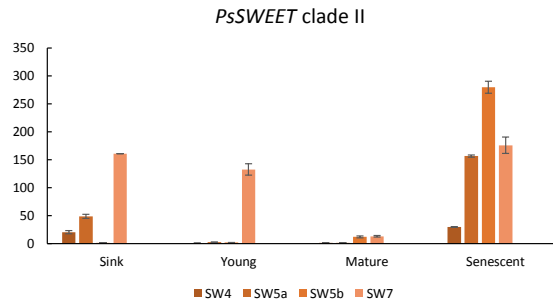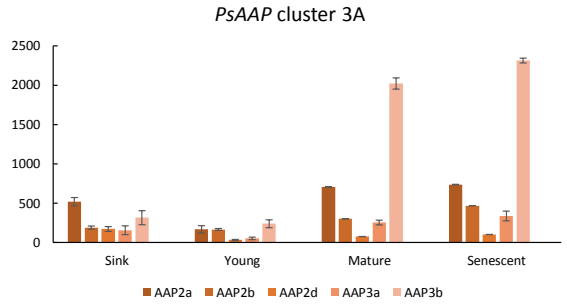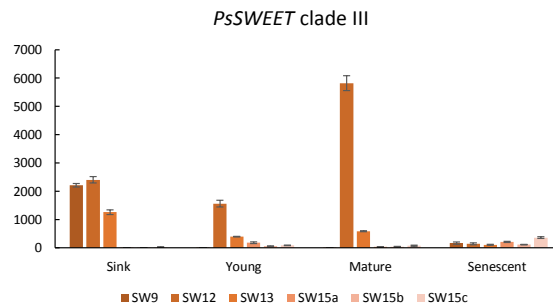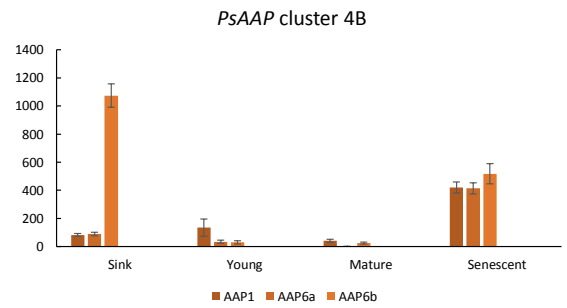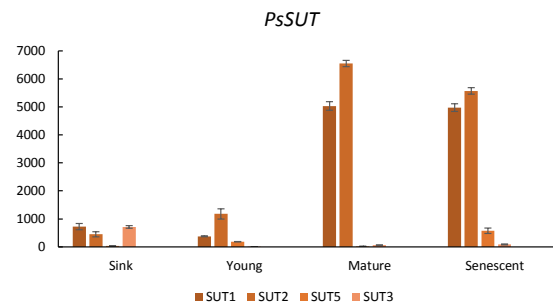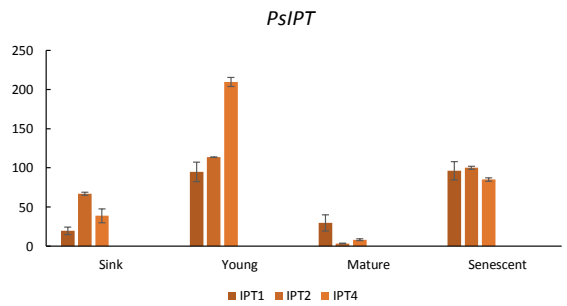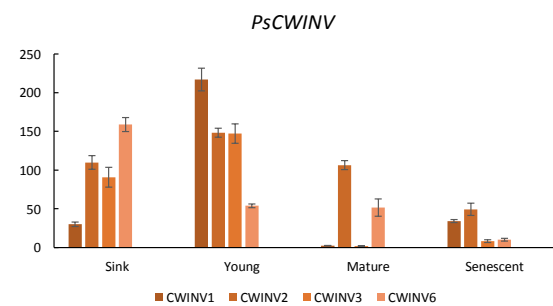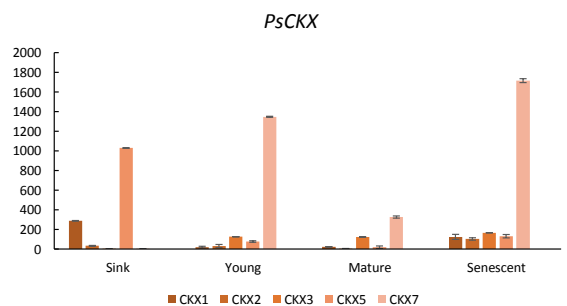

Supplement: Supplementary file 1 [file plants-08-00076-s001.zip › Fig 3 Bohatyr graphs leaves with y axes ticks.pdf]

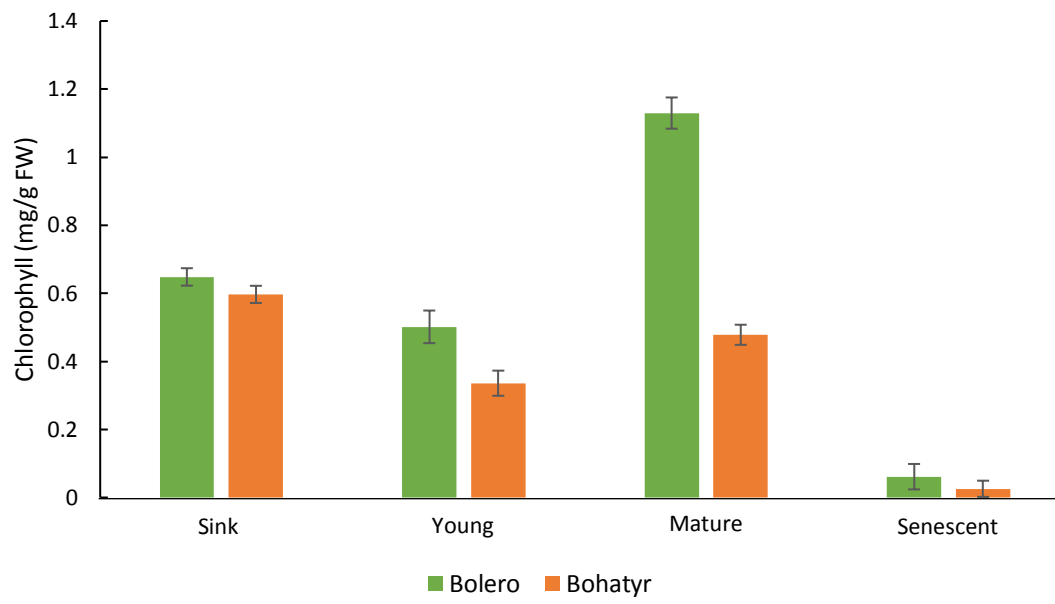

Supplement: Supplementary file 1 [file plants-08-00076-s001.zip › Fig 1 Chlorophyll with y axis ticks.pdf]

Relative expression (mean fold change)

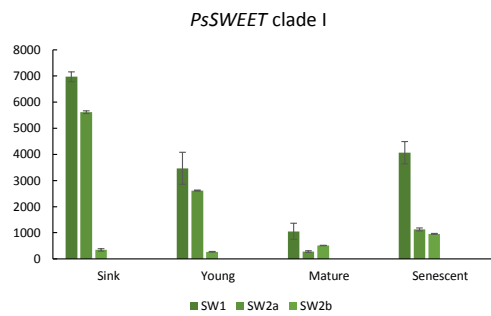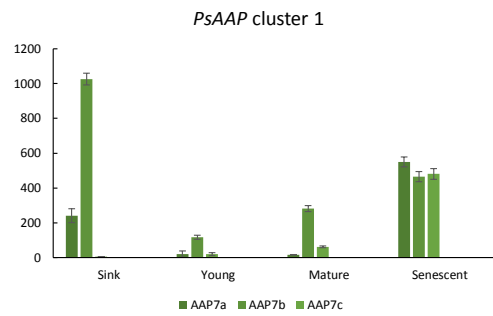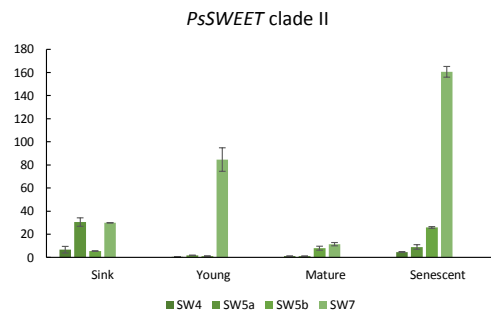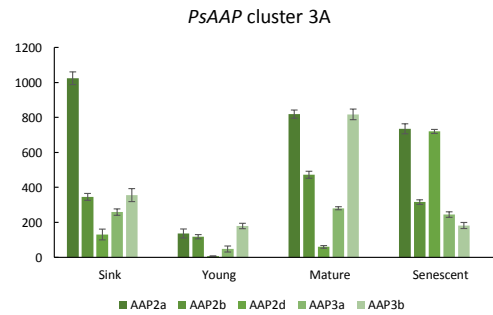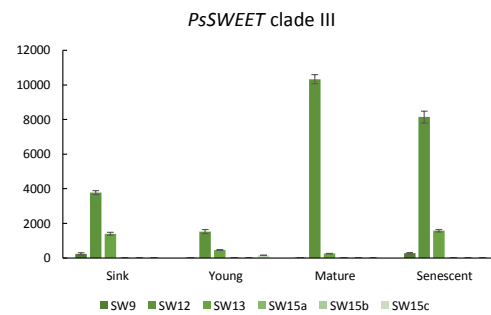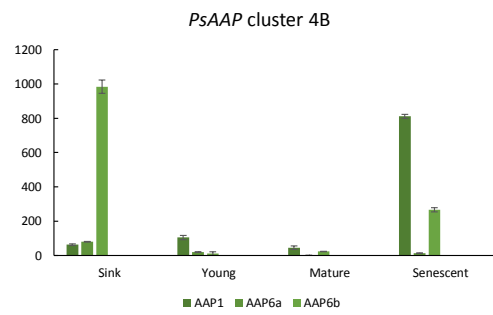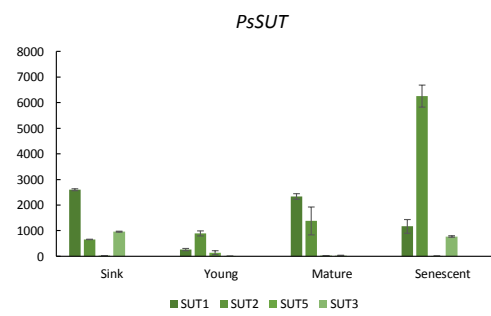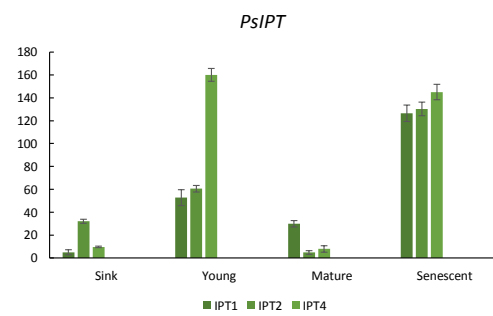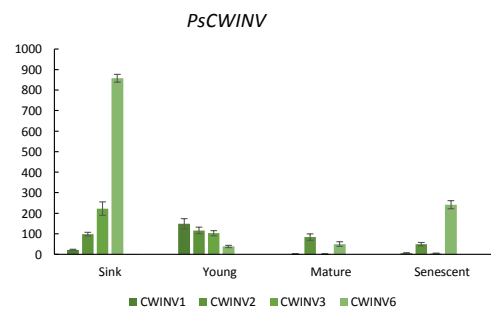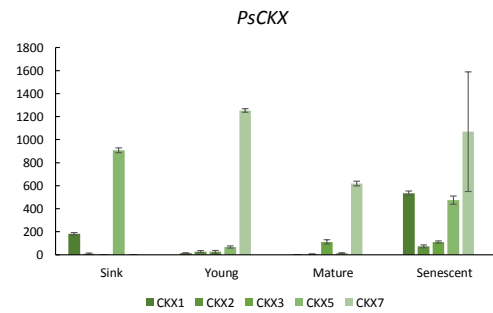

Supplement: Supplementary file 1 [file plants-08-00076-s001.zip › Fig 2 Bolero graphs leaves with y axes ticks.pdf]
